# Editorial: Non-tuberculous mycobacteria infections and COVID-19

**DOI:** 10.3389/fcimb.2025.1550277

**Published:** 2025-02-12

**Authors:** Vishwanath Venketaraman

**Affiliations:** Department of Basic Medical Sciences, Western University of Health Sciences, Pomona, CA, United States

**Keywords:** mycobacterial diseases, TB, NTM diseases, COVID-19, SARS-CoV2, non-tuberculous mycobacteria

## Introduction

Pulmonary diseases due to mycobacteria cause significant morbidity and mortality to human health. Mycobacterial lung infections are caused by mycobacteria, which include the causative agents of tuberculosis (TB) and leprosy. Nontuberculous mycobacteria (NTM) are also ubiquitous in soil, water, and food. They are usually harmless to people but for unknown reasons, NTM lung infections are becoming more prevalent in the developed world, including the United States, particularly in the Southwest (including southern California), Southeast, and Hawaii.

Recent epidemiological studies have shown the emergence of NTM species in causing lung diseases in humans. Although more than 170 NTM species are present in various environmental niches, only a handful, primarily *M. avium* complex and *M. abscessus*, have been implicated in pulmonary disease (Sebti et al., 2024). Following inhalation, both Mtb and NTM are phagocytosed by alveolar macrophages in the lungs. Subsequently, various immune cells are recruited from the circulation to the site of infection, which leads to granuloma formation. Although the pathophysiology of TB and NTM diseases share several fundamental cellular and molecular events, the host-susceptibility to Mtb and NTM infections are different. Striking differences also exist in the disease presentation between TB and NTM cases.

While NTM disease is primarily associated with bronchiectasis, this condition is rarely a predisposing factor for TB. Similarly, in Human Immunodeficiency Virus (HIV)-infected individuals, NTM disease presents as a disseminated, extrapulmonary form rather than as a pulmonary disease, which is seen in Mtb infection. Since NTM is causing lung diseases in humans along with COVID-19, and the interaction of these two morbidities has not been investigated much so far, our aim with this Research Topic, is to collect all the most recent data to aid the national health systems and health workers of the countries struck by NTM plus COVID–19 and to gather useful knowledge to tackle these infectious diseases together. Finally, laboratory diagnosis and treatment of both diseases are quite different.

In this comprehensive Research Topic Kuenstner et al. investigated cytokine expression in the samples from Temple University/Abilene Christian University (TU/ACU) study that had a prospective case control design with 201 subjects including 61 crohn’s disease (CD) patients and 140 non-CD controls. Findings from this study indicate that most subjects in the study samples had *Mycobacterium avium* ssp. *paratuberculosis* (MAP) infection and 8 of 9 subjects remained MAP positive one year later indicating persistent infection (Zhu et al.) While not identical, cytokine expression patterns in MAP culture positive CD patients in the TU/ACU study showed similarities (increased IL-17, IFNγ and TNFα) to patterns of patients with TB in other studies, indicating the possibilities of similar mechanisms of pathogen infection and potential strategies for treatment.

The accurate identification of the Mtb complex (MTBC) and different NTM species is crucial for the timely diagnosis of NTM infections and for reducing poor prognoses. Nucleotide matrix-assisted laser desorption/ionization time-of-flight mass spectrometry (MALDI-TOF-MS) has been extensively used for microbial identification with high accuracy and throughput. However, its efficacy for mycobacterium species identification has been less studied. The objective of Zhu et al’s study was to evaluate the performance of nucleotide MALDI-TOF-MS for mycobacterium species identification. The total correct detection rate of all 933 clinical mycobacterium isolates using nucleotide MALDI-TOF-MS was 91.64% (855/933), and mixed infections were detected in 18.65% (174/933) of the samples. The correct detection rates for *M. intracellulare*, *M. abscessus*, *M. kansasii*, *M. avium*, MTBC, *M. gordonae*, and *M. massiliense* were 99.32% (585/589), 100% (86/86), 98.46% (64/65), 94.59% (35/37), 100.00% (34/34), 95.65% (22/23), and 100% (19/19), respectively. For the identification of the MTBC*, M. intracellulare*, *M. abscessus*, *M. kansasii*, *M. avium*, *M. gordonae*, and *M. massiliense*, nucleotide MALDI-TOF-MS and Sanger sequencing results were in good agreement (k > 0.7). In conclusion, nucleotide MALDI-TOF-MS is a promising approach for identifying MTBC and the most common clinical NTM species.

Severe acute respiratory syndrome coronavirus-2 causes hyperinflammation and activation of coagulation cascade and, as a result, aggravates endothelial cell dysfunction. N-acetylcysteine and Sulodexide have been found to mitigate endothelial damage. The influence on coronary artery endothelial cells of serum collected after 4 ± 1 months from coronavirus infection was studied by Rajewska-Tabor et al. The concentrations of serum samples of interleukin 6, von Willebrand Factor, tissue Plasminogen Activator, and Plasminogen Activator Inhibitor-1 were studied. The cultures with serum of patients after coronavirus infection were incubated with N-acetylcysteine and Sulodexide to estimate their potential protective role. The blood inflammatory parameters were increased in the group of cultures incubated with serum from patients after coronavirus infection. Supplementation of the serum from patients after coronavirus infection with N-acetylcysteine or Sulodexide reduced the synthesis of interleukin 6 and von Willebrand Factor. No changes in the synthesis of tissue Plasminogen Activator were observed. N-acetylcysteine reduced the synthesis of Plasminogen Activator Inhibitor-1. N-acetylcysteine and Sulodexide increased the tPA/PAI-1 ratio. N-acetylcysteine may have a role in reducing the myocardial injury occurring in the post-COVID-19 syndrome. Sulodexide can also play a protective role in post-COVID-19 patients.

This Research Topic also includes research findings from Ma et al. and He et al. Ma et al. reported findings from their retrospective multicenter study conducted across 83 intensive care units (ICUs) in 16 cities in Sichuan, China. Critically ill patients diagnosed with heatstroke and lung infections were included in the study. Specimens from the lower respiratory tract were collected for microbiological testing, and the characteristics of the pathogens were described. A total of 462 patients diagnosed with heatstroke-related pulmonary infections were included, 134 patients (29.0%) tested positive for respiratory pathogens. The most frequently isolated strain was *Klebsiella pneumoniae* (34.3%), followed by *Escherichia coli* (28.4%), *Staphylococcus aureus* (20.9%). The results revealed that in the hyperthermic resistance group, there was a higher proportion of *Pseudomonas aeruginosa* [14(34.1%) vs 11(11.8%), *p*=0.002] and *Stenotrophomonas maltophilia* [4(9.8%) vs 1(1.1%), *p*=0.030] compared to the hyperthermic control group. And a higher proportion of *Staphylococcus aureus* [27(29.7%) vs 1(2.3%), *p*<0.001], were obtained during the earlier stages with elevated temperatures. Patients with *Klebsiella pneumoniae* (38.3 ± 1.9°C), *Staphylococcus aureus* (38.5 ± 2.2°C), and *Pseudomonas aeruginosa* (38.7 ± 1.9°C) exhibited a higher temperature environment. This study provides crucial insights into the lower respiratory tract pathogenesis of heatstroke patients, identifying key pathogens and their temperature-dependent characteristics, thus providing a foundation for future empirical treatment strategies in heatstroke.

He et al. investigated to investigated the diagnostic value of IL-17 detection in bronchoalveolar lavage fluid (BALF) and plasma samples from nonneutropenic patients with invasive pulmonary aspergillosis. A total of 281 patients were enrolled in this study, of which 62 had proven or probable IPA and the remaining 219 patients were controls. The plasma and BALF IL-17 levels were significantly higher in the IPA group compared with the control group. The plasma GM, plasma IL17, BALF GM, and BALF IL17 assays had sensitivities of 56.5%, 72.6%, 68.7%, and 81.2%, respectively, and specificities of 87.7%, 69.4%, 91.9%, and 72.6%, respectively. The sensitivity of IL17 in plasma and BALF was higher than that of GM. Plasma GM in combination with IL-17 increases the sensitivity but does not decrease the diagnostic specificity of GM testing. The diagnostic sensitivity and specificity of BALF GM combined with IL-17 for IPA in non-neutropenic patients were greater than 80% and there was a significant increase in sensitivity compared with BALF GM. Findings from this study indicate that the levels of IL-17 in the plasma and BALF were significantly higher in non-neutropenic patients with IPA. The sensitivity of plasma and BLAF IL-17 for diagnosing IPA in non-neutropenic patients was superior to that of GM. Combined detection of lavage fluid GM and IL17 significantly improves the diagnosis of IPA in non-neutropenic patients. The combined detection of GM and IL-17 in plasma also contributes to the diagnosis of IPA in patients who cannot tolerate invasive procedures.
